# Clinical Implications of Sodium Zirconium Cyclosilicate Therapy in Patients with Systolic Heart Failure and Hyperkalemia

**DOI:** 10.3390/jcm10235523

**Published:** 2021-11-25

**Authors:** Teruhiko Imamura, Akira Oshima, Nikhil Narang, Koichiro Kinugawa

**Affiliations:** 1 Second Department of Medicine, University of Toyama, Toyama 930-0194, Japan; brother0917jp@gmail.com (A.O.); kinugawa-tky@umin.ac.jp (K.K.); 2 Advocate Christ Medical Center, Oak Lawn, IL 60453, USA; nikhil.narang@gmail.com

**Keywords:** chronic kidney disease, potassium, hyperkalemia

## Abstract

Background: Sodium zirconium cyclosilicate (SZC), a newly introduced specific potassium binder, is introduced to treat hyperkalemia. However, the implications of SZC in up-titrating renin–angiotensin–aldosterone system inhibitors in patients with systolic heart failure remain unknown. Methods and Results: Patients with heart failure with left ventricular ejection fraction <50% and hyperkalemia who had completed 3-month SZC therapy were retrospectively included. Serum potassium levels, the dose of renin–angiotensin–aldosterone system inhibitors, and echocardiographic parameters during the 3-month SZC therapy as compared with the pretreatment 3-month period were investigated. A total of 24 patients (median 77 years old, 71% men, median left ventricular ejection fraction 41%) received a 3-month SZC therapy without any associated adverse events including hypokalemia. Compared with the pretreatment period, serum potassium levels decreased, doses of renin–angiotensin–aldosterone system inhibitors increased, and the left ventricular ejection fraction and plasma B-type natriuretic peptide levels improved following the 3-month SZC therapy (*p* < 0.05 for all). Conclusions: SZC may be a promising therapeutic option to improve hyperkalemia, indirectly allowing up-titration of renin–angiotensin–aldosterone system inhibitors and facilitating reverse remodeling in patients with heart failure with a left ventricular ejection fraction <50% and hyperkalemia.

## 1. Background

Hyperkalemia, which is in general defined as a serum potassium level above 5.0 mEq/L, is often encountered in patients with heart failure with reduced ejection fraction (HFrEF), owing to concomitant chronic kidney disease [1,2,3]. Its presence sometimes limits the up-titration of guideline-directed renin–angiotensin–aldosterone system inhibitors and indirectly affects long-term outcomes [4,5].

Recently, sodium zirconium cyclosilicate (SZC), a non-polymer zirconium silicate compound that exchanges sodium and hydrogen for potassium and ammonium ions in the gastrointestinal tract, was introduced to lower serum potassium levels [6]. However, its efficacy in patients with HFrEF, who should receive guideline-directed medications that can increase serum potassium levels, remains uncertain. A reduction in the incidence of hyperkalemia by SZC may indirectly allow for aggressive up-titration of guideline-directed potassium-preserving medications and facilitate reverse remodeling [7].

We investigated the trend in serum potassium levels, doses of renin–angiotensin–aldosterone system inhibitors, and echocardiographic parameters during a 3-month trial of SZC therapy in patients with HFrEF in real-world daily practice, compared with a 3-month pretreatment period.

## 2. Methods

### 2.1. Patient Selection

Patients with heart failure with a left ventricular ejection fraction (LVEF) <50%, who received SZC for 3 months or longer between July 2020 and May 2021 to treat hyperkalemia (defined as serum potassium level >5.0 mEq/L), were retrospectively included [8]. Patients dependent on hemodialysis and those taking sodium polystyrene sulfonate were not included. Informed consents were obtained from all participants and the present study was approved by the institutional ethical board beforehand.

### 2.2. Study Design

Patients were observed for 3 months before SZC therapy (pretreatment period) and 3 months following the initiation of SZC (treatment period on SZC). Baseline was defined as the time of SZC initiation. A primary endpoint was a trend in serum potassium level during the on-SZC period as compared with the pretreatment period. Secondary endpoints were trends in doses of renin–angiotensin–aldosterone system inhibitors, LVEF, plasma B-type natriuretic peptide (BNP) level, and renal function.

### 2.3. SZC Therapy

According to our institutional protocol, we initiated and continued SZC at a maintenance dose (5–15 g/day) to treat hyperkalemia. We considered terminating SZC for hypokalemia with serum potassium level <4.0 mEq/L.

### 2.4. Other Clinical Management

All patients received guideline-directed medical therapy. Medications were attempted to be initiated and up-titrated as tolerated considering patients’ clinical status, electrolytes including serum potassium level, and renal function.

### 2.5. Data Collection

Baseline demographic, echocardiographic, laboratory, and medication data obtained within 24 h before the SZC administration were retrieved. Estimated glomerular filtration ratio was calculated according to the Cockcroft–Gault method. Doses of beta-blocker were presented as carvedilol equivalents. Doses of angiotensin-converting enzyme inhibitors were presented as enalapril equivalents. Doses of mineralocorticoid receptor antagonists were presented as spironolactone equivalents. Data obtained 3 months before the SZC administration and 3 months following the SZC administration were also collected.

### 2.6. Statistics

Statistics were conducted using SPSS Statistics 22 (SPSS Inc., Armonk, IL, USA). Two-tailed *p*-values < 0.05 were considered statistically significant. All variables were assumed as nonparametric data considering the small sample size.

Trends in clinical parameters including serum potassium levels among 3 time points (pre 3 months, baseline, and post 3 months on SZC) were compared using Friedman’s test and a post-hoc Wilcoxon signed-rank test. Trends in the rate of medication prescription among 3 time points were compared using Qochran’s Q test and a post-hoc McNemar’s test. Changes in clinical parameters during the pretreatment period and those during the treatment period were compared by a Mann-Whitney U test.

## 3. Results

### 3.1. Baseline Characteristics

A total of 24 patients (median age 77 (68, 86) years old, 17 (71%) men, median LVEF 41% (32%, 46%)) received a 3-month SZC therapy to treat hyperkalemia and were included in this study (Table 1). The baseline serum potassium level was 5.5 (5.2, 5.8) mEq/L. SZC was initiated at a maintenance dose in all patients (5–15 mg/day). The doses were adjusted within the maintenance range during the treatment period, targeting a serum potassium level <5.0 mEq/L.

During the SZC therapy, no patients had SZC-related adverse events including edema, nasopharyngitis, and upper respiratory tract infection. For renin–angiotensin–aldosterone system inhibitors, all patients took angiotensin-converting enzyme II inhibitors. Nobody received angiotensin receptor antagonists or sacubitril/valsartan. All patients were educated to obey the potassium restriction diets (<2000 mg/day of potassium intake).

### 3.2. Before SZC Initiation

**Figure 1:**
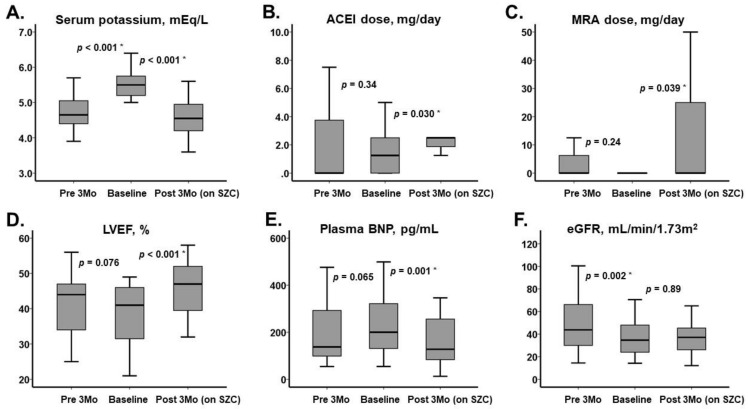
Trends of major clinical parameters including serum potassium level at 3 time points (pre 3 months, baseline, and post 3 months on SZC). (**A**) Serum potassium, (**B**) ACEI dose, (**C**) MRA dose, (**D**) LVEF, (**E**) Plasma BNP, (**F**) eGFR. ACEI, angiotensin-converting enzyme inhibitor; MRA, mineralocorticoid receptor antagonist; LVEF, left ventricular ejection fraction; BNP, B-type natriuretic peptide; eGFR, estimated glomerular filtration ratio. * *p* < 0.05 by Wilcoxon signed-rank test.

**Figure 2:**
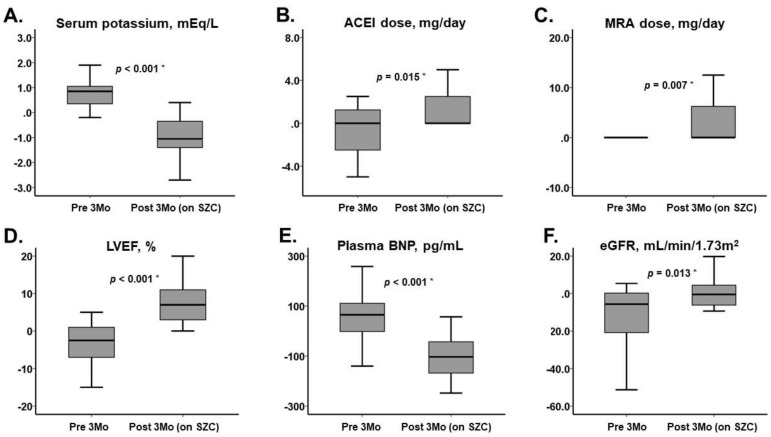
Changes in major clinical parameters during pretreatment period and treatment period on SZC. (**A**) Serum potassium, (**B**) ACEI dose, (**C**) MRA dose, (**D**) LVEF, (**E**) Plasma BNP, (**F**) eGFR. Abbreviations as in Figure 1. * *p* < 0.05 by Mann-Whitney U test.

Trends and changes in major clinical parameters are summarized in Figure 1; Figure 2, respectively. Trends in the rate of medication prescription are summarized in Table 2. Three months before SZC initiation, the serum potassium level was 4.7 (4.4, 5.1) mEq/L. Median doses of carvedilol, enalapril, and spironolactone were 2.5 (0, 8.75) mg, 0 (0, 3.75) mg, and 0 (0, 6.25) mg, respectively. eGFR was 43.8 (30.0, 66.2) mL/min/1.73 m^2^, plasma BNP was 138 (99, 293) pg/mL, and LVEF was 44% (34%, 47%).

During the 3-month pretreatment period, serum potassium level increased to 5.5 (5.2, 5.8) mEq/L. Doses of heart failure medications and their prescription rates remained unchanged (*p* > 0.05 for all). LVEF and plasma BNP remained unchanged. eGFR worsened to 34.7 (24.0, 48.0) mL/min/1.73 m^2^. The New York Heart Association functional class (I/II/III/IV) worsened from 0/21/3/0 to 0/16/6/2 (*p* = 0.020).

### 3.3. After SZC Initiation

Following the 3-month SZC therapy, the serum potassium level decreased to 4.6 (4.2, 5.0) mEq/L. Prescription rates of enalapril and spironolactone increased significantly (*p* < 0.05 for both). Doses of carvedilol remained unchanged (*p* = 0.087), whereas doses of enalapril and spironolactone increased up to 2.5 (1.9, 2.5) mg and 0 (0, 25) mg, respectively (*p* = 0.030 and *p* = 0.039). LVEF and plasma BNP improved significantly (*p* < 0.001 and *p* = 0.001, respectively). eGFR remained unchanged (*p* = 0.89). The New York Heart Association functional class (I/II/III/IV) tended to improve from 0/16/6/2 to 5/15/4/0 (*p* = 0.16). Six-minute walk distance increased significantly during the treatment period (from 325 (285, 351) m to 341 (295, 368) m) (*p* = 0.038).

## 4. Discussion

We investigated the trend in clinical parameters, including serum potassium levels, following a 3-month SZC therapy in patients with HFrEF and hyperkalemia. Following the 3-month SZC therapy, overall serum potassium levels decreased, doses of renin–angiotensin–aldosterone system inhibitors were up-titrated, and cardiac function improved as demonstrated by positive respective changes in LVEF, plasma BNP levels, and functional capacity.

### 4.1. Impact of SZC on Hyperkalemia

The HARMONIZE phase III double-blind randomized control study found that a 30-day 5 or 10 g/day SZC therapy maintained normokalemia in most of the recipients (58.6% and 77.3%, respectively) [8]. Of note, this trial included only 19% of heart failure patients. The impact of SZC may differ in patients with HFrEF, who often receive potassium-preserving medications. In this real-world observational study, serum potassium levels decreased significantly following 3 months of SZC therapy, by approximately −0.9 mEq/L in the setting of heart failure therapies, which can increase potassium.

SZC was initiated and continued at a maintenance dose in all patients. In our previous study, a proportion of heart failure patients experienced hypokalemia by SZC therapy during the loading dosage [9]. Of note, advanced age, reduced renal function, and the concomitant use of diuretics were associated with SZC-related hypokalemia [9]. Hypokalemia increases the risk of malignant arrhythmias in heart failure patients. We observed no cases of patients with serum potassium levels <3.5 mEq/L during the SZC therapy.

### 4.2. Impact of SZC to Up-Titrate Renin–Angiotensin–Aldosterone System Inhibitors

Renin–angiotensin–aldosterone system inhibitors can commonly increase serum potassium levels, which can limit their implementation in patients with chronic kidney disease [4,5]. Initiation and up-titration of these therapies to target doses that confer the greatest mortality benefit are often not reached in patients with HFrEF due to these side effects. There is a clear therapeutic need in contemporary practice to address the issue of inertia in titrating medical therapies for heart failure patients, with one of the most common reasons cited by practitioners being the perceived risk of hyperkalemia.

Following the reduction of hyperkalemia by SZC therapy, renin–angiotensin–aldosterone system inhibitors were up-titrated in this study. Prospective randomization trials are needed, however, to ascertain the impact of these therapies on key clinical endpoints. LVEF and plasma BNP levels remained unchanged during the pretreatment period, whereas these parameters likely improved in the post-treatment period due to the up-titration of heart failure-specific therapies [10]. In this analysis, we observed that SZC therapy improves hyperkalemia and may allow for a higher likelihood of up-titration of renin–angiotensin–aldosterone system inhibitors, which would indirectly have facilitated reverse cardiac remodeling.

### 4.3. Limitations

This is a proof-of-concept study with a small sample size. This is a retrospective observational analysis; we cannot infer causality. Dose adjustment of medical therapies was decided on at the discretion of the attending physicians. No patients received sacubitril/valsartan. We measured plasma BNP levels but not NTpro-BNP levels. The applicability of our findings to those receiving sacubitril/valsartan remains for future concern. We believe that SZC-incorporated sacubitril/valsartan therapy would further increase the clinical advantage.

## 5. Conclusions

SZC may be a promising therapeutic tool to improve hyperkalemia, allowing up-titration of renin–angiotensin–aldosterone system inhibitors and facilitating cardiac reverse remodeling in patients with heart failure with LVEF <50% and hyperkalemia.

## Figures and Tables

**Table 1:** Baseline characteristics.

	*n* = 24
Demographics	
Age, years	77 (68, 86)
Men	17 (71%)
Body mass index, kg/m^2^	21.7 (19.1, 23.0)
Comorbidity	
Hypertension	16 (67%)
Atrial fibrillation	7 (29%)
Diabetes mellitus	9 (38%)
Ischemic heart disease	11 (46%)
Hemodynamics	
Systolic blood pressure, mmHg	119 (107, 138)
Heart rate, bpm	77 (67, 88)
Medication	
Beta-blocker, mg/day	2.5 (0, 6.25)
Angiotensin-converting enzyme inhibitor, mg/day	1.25 (0, 2.5)
Mineralocorticoid receptor antagonist, mg/day	0 (0, 0)
Furosemide, mg/day	10 (0, 20)
Laboratory data	
Hemoglobin, g/dL	11.7 (10.9, 12.8)
Serum potassium, mEq/L	5.5 (5.2, 5.8)
Serum sodium, mEq/L	137 (134, 140)
eGFR, mL/min/1.73 m^2^	34.7 (24.0, 48.0)
Serum total bilirubin, mg/dL	1.2 (0.8, 1.5)
Serum C-reactive protein, mg/dL	0.6 (0.2, 0.9)
Plasma B-type natriuretic peptide, pg/mL	200 (131, 322)
Echocardiography	
Left ventricular end-diastolic diameter, mm	51 (45, 58)
Left ventricular ejection fraction, %	41 (32, 46)
Left atrial diameter, mm	41 (35, 44)
Six-minute walk distance, m	325 (285, 351)

eGFR, estimated glomerular filtration ratio. Continuous variables are presented as median and interquartile. Categorical variables are presented as number and percentage.

**Table 2:** Trend in the rate of medication prescription.

	Pre 3 Months	Baseline	Post 3 Months	*p* Value
Beta-blocker	15 (63%)	17 (71%)	17 (71%)	0.37
Angiotensin converting enzyme II inhibitor	10 (42%)	14 (58%)	18 (75%) ^†^	0.008 *
Mineralocorticoid receptor antagonist	5 (21%)	5 (21%)	7 (27%) ^†^	0.008 *
Loop diuretics	11 (46%)	13 (54%)	15 (63%)	0.14

* *p* < 0.05 by Qochran’s Q test. ^†^ *p* < 0.05 by McNemar’s test as compared to the prior timing.

## Data Availability

Data are available from the corresponding author upon reasonable request.
